# Responses to Voluntary Isocapnic Hyperpnea in Normoxia and Hypoxia: Insights from Blood Gas Analysis

**DOI:** 10.3390/biology14091207

**Published:** 2025-09-07

**Authors:** Tomasz Kowalski

**Affiliations:** Department of Physiology, Institute of Sport—National Research Institute, 00-189 Warsaw, Poland; tomasz.kowalski@insp.pl

**Keywords:** blood gas analysis, blood gasometry, BGA, pH, respiratory muscle training, voluntary isocapnic hyperpnea, VIH, hypoxia, altitude

## Abstract

Voluntary Isocapnic Hyperpnea (VIH), a respiratory muscle training method, is assumed to stabilize blood CO_2_ levels during increased ventilation, potentially supporting cellular homeostasis. The study aimed to empirically validate the concept and determine whether VIH effectively preserves key blood gas indices across different ambient oxygen levels in various populations. Two cross-sectional experiments (longitudinal in normoxia in highly trained athletes, *n* = 9 and single session in severe hypoxia of 4200 m above sea level in healthy and active participants, *n* = 18) were performed. Paired Bayesian *t*-tests and repeated measures analysis of variance were used to compare values of hydrogen ion concentration (pH), bicarbonate ion (HCO_3_^−^), partial pressure of oxygen (pO_2_), and partial pressure of carbon dioxide (pCO_2_) before and after VIH sessions. Except for pO_2_ (BF_10_ = 1.596 to 7.986), there were no meaningful differences in the analyzed variables before and after VIH in normoxia (BF_10_ = 0.322 to 0.490). These findings remained consistent for different familiarization and training statuses of participants, as well as sessions’ length and intensity. The likelihood of differences in pH, pO_2_, and pCO_2_ in hypoxia was supported by BF_10_ values between 1.349 and 6.304. No between-sex differences were observed. Our observations highlight the physiological robustness of VIH in maintaining blood gas and pH equilibrium in normoxia, with potential implications for supporting cellular acid–base homeostasis and mitochondrial function. In severe hypoxia, VIH was associated with changes in multiple analyzed variables, suggesting the need for caution, along with increased requirements for protocol individualization and monitoring.

## Simple Summary

Voluntary Isocapnic Hyperpnea (VIH), one of the most popular respiratory training methods, effectively maintains blood gas and pH homeostasis in normoxia in healthy adults. However, VIH in severe hypoxia was associated with changes in pH and pO_2_, suggesting the need for caution, along with increased requirements for protocol individualization and monitoring. Further research is undoubtedly warranted to investigate different VIH systems and to include vulnerable populations, such as older adults and individuals with underlying health conditions.

## 1. Introduction

Respiratory muscle training (RMT) is a targeted conditioning approach designed to enhance the strength and endurance of the muscles involved in breathing. RMT can be effectively applied in both clinical and athletic populations to enhance breathing efficiency and performance. In patients with respiratory conditions such as COPD or heart failure, RMT helps reduce dyspnea and improve exercise tolerance by strengthening weakened inspiratory and expiratory muscles [1,2]. In older adults, it may improve functional capacity and quality of life [3]. For athletes, RMT can improve endurance performance, increase ventilatory efficiency, delay the onset of respiratory muscle fatigue, and improve oxygen delivery during high-intensity efforts [4,5]. The primary mechanisms behind its effectiveness include improved diaphragmatic function, reduced work of breathing, enhanced neuromuscular coordination, and mitigation of respiratory metaboreflex [6,7]. The most commonly used methods are pressure threshold loading (PTL) using resistance devices and endurance-oriented voluntary isocapnic hyperpnoea (VIH), both of which systematically overload the respiratory muscle to stimulate adaptation [4].

Although both methods vary, they typically yield similar outcomes in patients, healthy individuals, and athletes [1,8,9]. Notably, PTL was associated with an imbalance in blood gasometry, an increase in lactate, and reports of headaches and dizziness [10]. VIH, on the other hand, employs rebreathing bags, which should allow for stable blood oxygen and carbon dioxide (CO_2_) levels and limit negative symptoms often associated with RMT. Multiple VIH devices, such as Spirotiger (Idiag AG, Zurich, Switzerland) or ISO-BWB (Isocapnic Technologies Inc., Kelowna, Canada), are widely applied in RMT practice and research. VIH is considered “isocapnic”, as it purposefully prevents hypocapnia that typically accompanies hyperpnea by balancing vigorous ventilation with CO_2_ addition, a feature that distinguishes it from simple hyperventilation [4]. Given that enzymatic function and cellular metabolism are pH-sensitive, the maintenance of systemic acid–base balance during respiratory interventions such as VIH could play a critical role in supporting cellular homeostasis. Importantly, cellular responses to blood gases and acid–base disturbances often involve the activation of adaptive stress pathways such as autophagy, endoplasmic reticulum stress responses, and apoptosis [11,12]. Stabilization of blood pH and pCO_2_ during VIH may help limit the activation of these pathways, supporting cellular resilience by maintaining an optimal intracellular environment for protein folding, redox balance, and mitochondrial integrity [13]. Moreover, extracellular pH fluctuations are known to influence multiple cell signaling cascades, including mitogen-activated protein kinases (for example, MAPKs) pathways, which are critically involved in stress response, metabolism, and inflammation [14,15]. Finally, immune cells, particularly macrophages and T lymphocytes, exhibit pH-sensitive behavior, with acidotic conditions affecting their function, survival, and proliferation [16]. Overall, disturbances in blood gasometry may augment malaise and would encourage reconsideration of VIH safety and feasibility.

However, the premise regarding isocapnic properties on VIH has been introduced as a general concept, but its empirical validation in peer-reviewed literature remains absent. The classic approach for maintaining isocapnia was originally applied by rebreathing air enriched with a precisely calculated CO_2_ amount [17]. However, during VIH, a different solution remains widespread. Namely, the exhaled air is stored in a dedicated bag to be immediately rebreathed again. Naturally, it contains increased CO_2_ concentrations, but it remains unknown if this method effectively minimizes homeostasis disturbance. Basic biophysics [18,19] suggests that the outcome may depend on individual chemosensitivity, the volume and tightness of the rebreathing circuit, and the duration and intensity of the VIH session. Therefore, this study seeks to move beyond the conceptual framework and subject the commonly accepted model to empirical scrutiny. Two complementary experiments with blood gas analysis (BGA) were conducted. Experiment 1 examined the physiological responses to VIH in normoxic conditions across a 6-week RMT intervention in well-trained athletes. Experiment 2 was held in hypoxic conditions to assess whether oxygen availability influences the maintenance of blood gas homeostasis during VIH in healthy subjects. Together, these experiments aimed to provide a more comprehensive understanding of the applicability of VIH across varying ambient oxygen levels in distinct populations.

## 2. Materials and Methods

The study protocol was reviewed and approved by the Ethics Committee of the Institute of Sport–National Research Institute, Warsaw, Poland (approval no. KEBN-23-78-TK from 17 February 2023 for Experiment 1 and approval no. KEBN-24-92-TK from 4 March 2024 for Experiment 2). Written informed consent was obtained from all participants prior to their involvement. All procedures were conducted in accordance with the principles outlined in the Declaration of Helsinki. Both experiments took place in the laboratories of the Institute of Sport–National Research Institute, Warsaw, Poland. The STROBE (Strengthening the Reporting of Observational Studies in Epidemiology) guidelines were followed wherever applicable [20].

### 2.1. Applied Devices and Procedures

**Figure 1:**
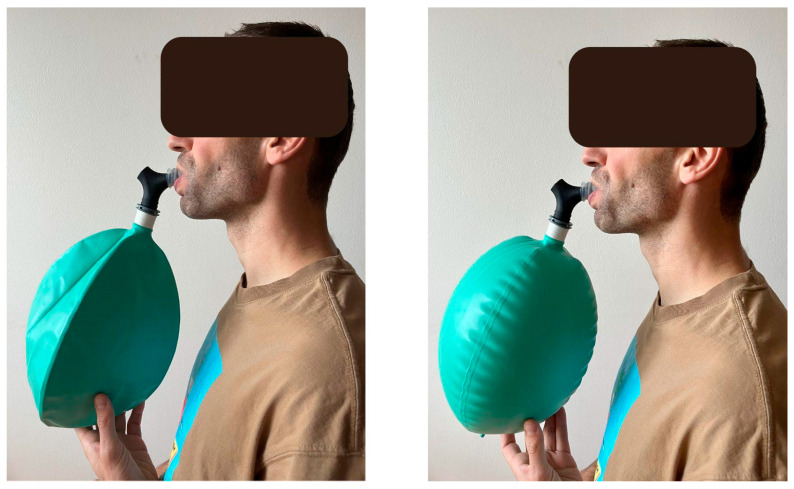
Presentation of Voluntary Isocapnic Hyperpnea (VIH). Adapted from Kowalski et al. (2024) [9].

Isocapnic BreathWayBetter devices (Isocapnic Technologies Inc., Kelowna, BC, Canada) with 6 L bags were used in both experiments (see Figure 1). This is a mobile device that requires no additional calibration. Dedicated Isocapnic App was used to guide the rhythm of breathing and track the length of the session. During VIH, the participants were instructed to employ diaphragmatic breathing and limit movement of the upper chest and shoulders. BGA was performed using a Radiometer™ ABL90 FLEX blood gas analyzer (Radiometer Medical ApS, Brønshøj, Denmark). The following parameters were measured: hydrogen ion concentration (pH), bicarbonate ion (HCO_3_^−^), partial pressure of oxygen (pO_2_), and partial pressure of carbon dioxide (pCO_2_). All the indices were measured in doubles, with 45 µL capillary blood samples taken from the fingertip. The analyses were performed immediately after sampling by an experienced and skilled technician, following the manufacturer’s guidelines. Participants’ height was measured using a free-standing digital stadiometer (seca 274, seca GmbH & Co. KG, Hamburg, Germany). In experiment 1, body mass was measured with a body composition analyzer (InBody 770, InBodyUSA, Cerritos, CA, USA). In experiment 2, body mass was measured with dual-energy X-ray absorptiometry (Lunar Prodigy Pro DXA, GE Healthcare, Chicago, IL, USA).

### 2.2. Experiment 1—VIH in Normoxia in Well-Trained Athletes

This study presents a secondary outcome analysis derived from data collected in a randomized controlled trial, originally registered at ClinicalTrials.gov as NCT05936398. Nine well-trained triathletes (4 females: age 29.5 ± 8.8, body mass 58.4 ± 1.3, height 166.7 ± 3.9; 5 males: age 30.8 ± 4.8, body mass 77.2 ± 4.6, height 185.0 ± 6.1), recruited via individual invitations, completed the 6-week RMT intervention. In addition to specific training, they performed a VIH session every second day. Progression overload, based on session length and breathing frequency, was applied. The detailed scheme of the intervention is presented in Table 1. All participants were classified as Tier 3 or Tier 4 athletes according to the Participant Classification Framework, indicating highly trained or elite status [21]. Inclusion criteria were a valid medical certificate for triathlon competition, no prior experience with RMT, a minimum of six years of triathlon training, an average weekly training volume exceeding 12 h over the past six weeks, and a performance level equivalent to at least a medal finish at a national multisport championship within the last two years. Exclusion criteria were any chronic or acute medical conditions within the past three months, or any ongoing medication or allergic reaction. For female participants, hormonal contraception use and time since last menstruation were recorded. All participants were in a base training phase, 10–14 weeks into the preparatory period and several months before the competitive season.

Before the intervention, participants underwent thorough familiarization with the supervised sessions. Across the program, the participants performed 3 monitored training sessions in the laboratory setting, in week 1 (session 3), week 4 (session 14), and week 6 (session 21). As presented in Table 1, these sessions varied in length and breathing frequency (5–20 min and 20–26 breaths * min^−1^). Moreover, participants differed in their level of familiarization and RMT status between the first and last monitored sessions. All the participants were adapted to the testing environment settings due to their previous testing experiences. The blood for BGA was sampled immediately before and after VIH sessions. The participants remained seated for 15 min before and during the measurements and VIH. Repeatable testing conditions were provided: altitude was 87 m above sea level (FiO2  =  20.8%), temperature from 20.7 °C to 22.1 °C, and humidity from 44% to 56%. The measurements were conducted at the same time between 8:30 and 10:30 a.m. to mitigate the impact of physiological diurnal variations.

### 2.3. Experiment 2—VIH in Severe Hypoxia

Experiment 2 involved healthy and active participants, recruited with convenience sampling. Inclusion criteria were excellent health, age between 18 and 39 years of age and meeting the World Health Organization’s basic physical activity recommendations, i.e., weekly engaging in 150–300 min of moderate-intensity aerobic activity or 75–150 min of vigorous-intensity aerobic activity, or an equivalent combination of both [22]. Exclusion criteria were hypoxia exposure during last 3 months, any chronic or acute medical conditions within the past three months, any ongoing medication or allergic reaction, pregnancy, smoking. All the participants were born at sea level. The total required sample size was calculated with G* Power (version 3.1.9.6; Dusseldorf, Germany), with the level of significance set at α = 0.05, power (1 − β) = 0.95, and effect size ƒ = 0.5 (ANOVA with repeated measures, within-between interaction, 2 groups, 2 measurements). The required total sample size, as determined by the calculations, was 16 participants. A total of 18 participants completed the experiment and were included in the analysis (age 28.6 ± 6.4, 8 females, 10 males).

After 2 familiarization sessions, the participants performed a single VIH session (5 min, 20 breaths * min^−1^) in normobaric hypoxia of 4200 m above sea level (FiO2  =  12.8%). After thorough medical screening, the participants entered a hypoxic chamber (Air Sport, Międzyzdroje, Poland) and remained seated for the whole time. Temperature (approximately 21 °C), humidity (45–50%), and oxygen and carbon dioxide concentrations were centrally controlled and kept steady throughout the exposure. The VIH session was performed 1 h after the entrance. The blood for BGA was collected immediately before and after the VIH session. The relevant safety procedures to minimize risk to participants were applied (constant medical supervision, oxygen saturation, and blood pressure monitoring, screening for acute mountain sickness symptoms with the Lake Louis scale).

### 2.4. Statistical Analyses

Data was checked for normality using the Shapiro–Wilk tests and visual assessments of Q–Q plots. In experiment 1, considering the sample size, the analysis was performed using paired Bayesian *t*-tests (Cauchy: 0, 0.707) to compare BGA measurements before and after VIH sessions. For each comparison, the Bayes Factor (BF_10_) was calculated to quantify evidence for the alternative hypothesis relative to the null. In experiment 2, both Bayesian *t*-tests (Cauchy: 0, 0.707) and repeated measures analysis of variance (ANOVA) were used. Homogeneity of variance was assessed with Levene’s test. For significant effects, post hoc Bonferroni correction was applied. The effect size was calculated as partial eta squared (ηp^2^) and omega squared (ω^2^). The values of approximately 0.01, 0.06, and 0.14 are conventionally interpreted as small, medium, and large effect sizes, respectively. A significance level of *p* < 0.05 was used. JASP statistical package (version 0.17.1, JASP Team, Amsterdam, The Netherlands) and was used for the analyses.

## 3. Results

### 3.1. Experiment 1

The Bayesian paired samples *t*-tests revealed anecdotal to moderate evidence in favor of a difference between pO_2_ before and after VIH (conventionally significant differences). However, the results support the conclusion that there were no meaningful differences before and after VIH for pH, HCO_3_^−^, and pCO_2_ (conventionally no significant differences). The estimation error for BF_10_ was minimal (under 0.01% for all analyses), suggesting a high degree of reliability in the resulting evidence assessment. Full results are presented in Table 2. This section may be divided by subheadings. It should provide a concise and precise description of the experimental results, their interpretation, as well as the experimental conclusions that can be drawn.

### 3.2. Experiment 2

The Bayesian paired samples *t*-tests revealed anecdotal to moderate evidence in favor of a difference between pH, pO_2_, and pCO_2_ before and after VIH (conventionally significant differences). The estimation error for BF_10_ was minimal (under 0.024% for all analyses), suggesting a high degree of reliability in the resulting evidence assessment. Full results are presented in Table 3.

Since the sample size allowed for ANOVA, repeated measures analysis with additional sex interaction (time and time * sex) was conducted. Clear significant differences were identified in pH (F = 9.538, *p* = 0.007, ηp^2^ = 0.373, ω^2^ = 0.193), and pO_2_ (F = 5.948, *p* = 0.027, ηp^2^ = 0.271, ω^2^ = 0.112). Effects for HCO_3_^−^ and pCO_2_ were on the verge of statistical significance (*p* = 0.056 and *p* = 0.068, respectively, large effect sizes for both variables). No significant differences were observed for time * sex interaction. However, the effect for HCO_3_^−^ was on the verge of statistical significance (F = 3.747, *p* = 0.071, ηp^2^ = 0.190, ω^2^ = 0.038) with a larger post-VIH increase in females. The exact significance levels for time * sex interactions in the remaining variables were as follows: *p* = 0.335 for pH, *p* = 0.326 for pO_2_, and *p* = 0.719 for pCO_2_.

## 4. Discussion

The study aimed to determine whether the RMT method, based on VIH, effectively preserves key blood gas indices across different ambient oxygen levels in various populations. Our findings indicate that, except for pO_2_, there are no meaningful differences in BGA variables before and after VIH in normoxia. These findings remain robust for different familiarization and training statuses of participants, as well as sessions’ length and intensity. Observations in hypoxia were less conclusive, suggesting the influence of VIH on pH and pO_2_, while the evidence remained equivocal regarding changes in pCO_2_ and HCO_3_^−^. No between-sex differences were detected.

The blood gases imbalances may activate stress pathways like autophagy, endoplasmic reticulum stress, and apoptosis [11,12]. Additionally, extracellular pH changes affect key signaling pathways and immune cell function [14,15,16]. However, the maintenance of systemic pH, HCO_3_^−^, and pCO_2_ observed during VIH suggests that the described mechanisms may remain relatively unperturbed in normoxia, potentially preserving homeostatic signaling dynamics under varying oxygen conditions and VIH sessions’ parameters. In normoxia, although VIH maintains isocapnia, it was proven to induce fluctuations in capillary blood pO_2_, especially in less familiarized participants and during longer and more intense sessions. Such periods of transient hyperoxia may lead to an increased production of reactive oxygen species [23,24]. Moreover, rapid shifts between normoxia and hyperoxia can disrupt oxygen-sensing pathways, contributing to endothelial stress and inflammatory responses [25,26]. However, controlled hyperoxic exposures have also been associated with enhanced oxygen delivery and may transiently improve aerobic metabolism and tissue oxygenation [27,28]. Although the research is not conclusive, some studies suggest that exposure to short periods of elevated oxygen levels may positively influence athletic performance [29,30]. Thus, while pO_2_ fluctuations during VIH may carry physiological costs, they also may offer adaptive or performance-related benefits depending on the context and population. Importantly, although the VIH-induced increases in pO_2_ seem likely, these values remain in the well-established clinical ranges [31]. In our study, the post-session increased pO_2_ means remained between 75 and 85 mmHg, and the reference of Radiometer™ ABL90 FLEX blood gas analyzer used to perform assays is up to 100 mmHg. Therefore, the observed changes appear clinically insignificant, supporting the safety and feasibility of VIH in healthy individuals. Moreover, Iqbal et al. (2025) examined a wide array of serum biomarkers to assess respiratory muscle damage following volitional hyperpnea in healthy young men. Only slow skeletal troponin was elevated 24 h post-hyperpnea compared to control, suggesting only a small possible respiratory muscle damage, while muscle creatine kinase and fast skeletal troponin showed no change [32]. Notably, greater caution is advised for vulnerable populations, such as patients or the elderly.

However, our observations in hypoxia highlight potential safety concerns associated with disturbances in homeostasis. Previously, hyperpnoea under hypoxic conditions was typically investigated during whole-body exercise [33]. This might be the first study regarding hyperpnoea as a distinct activity. The presented findings suggest that, in severe hypoxia, VIH may alter blood gas homeostasis, warranting careful monitoring and individualized protocols. Importantly, VIH typically introduces respiratory muscle fatigue [34,35]. At the same time, hypoxic exposure influences ventilatory response and leads to increased respiratory demands [36,37]. The coupling of both phenomena has not been investigated. Speculatively, one may enhance the other. Next, to improve respiratory muscle function, VIH may take up to 40 min in healthy individuals [38]. In our study, we investigated the influence of relatively short, 5 minute sessions. The observed changes in BGA were more pronounced compared to noteworthy longer sessions (i.e., 20 min of VIH) analyzed in normoxia. Consequently, VIH in severe hypoxia requires more caution and should be adapted to individual tolerance levels and include close physiological monitoring. Importantly, this consideration is not limited to healthy, young, and active populations. Globally, over 500 million people live in hypoxic environments, above 1500 m asl [39]. According to the WHO Chronic Respiratory Diseases Programme, approximately 12.5% of the world population may be affected by respiratory diseases. Simultaneously, the global senior (over 65 years old) population has reached 1 billion people and is rapidly increasing. Overall, individuals from vulnerable groups may be adversely affected by the influence of VIH under hypoxic conditions. A reconsideration of VIH application in patients and older adults living at altitude seems warranted.

While the findings provide valuable insights, the presented research has some limitations. First, the sample size in experiment 1 was small. Consequently, a replication study seems warranted to confirm the presented findings. Moreover, the sample size did not permit separate Bayesian analyses by sex. This may limit the interpretation of the results, as potential sex-related differences could not be examined. Furthermore, the investigation was based on one device only, and the outcomes should not be extrapolated to the whole range of devices used for VIH. Finally, BGA from capillary blood may be less accurate than venous sampling due to variability in peripheral perfusion and local tissue metabolism, which may affect the reliability of pO_2_ measurements. Notably, the outcomes from the experiments should not be directly compared, as they involved different populations. Further research is undoubtedly warranted to include different devices and rebreathing bag volumes. Finally, specific elderly and patient populations should be investigated, as they are most sensitive to homeostasis disturbances, and potential imbalance in blood gasometry may lead to worse symptoms compared to the younger, active, and healthy cohorts [40].

## 5. Conclusions

Our observations highlight the physiological robustness of VIH in maintaining blood gas and pH equilibrium in normoxia in healthy adults, with potential implications for supporting cellular acid–base homeostasis and mitochondrial function. VIH in severe hypoxia was associated with changes in pH and pO_2_, suggesting the need for caution, along with increased requirements for protocol individualization and monitoring. Further research is undoubtedly warranted to investigate different VIH systems and to include vulnerable populations, such as older adults and individuals with underlying health conditions.

## Figures and Tables

**Table 1:** The Voluntary Isocapnic Hyperpnoea (VIH) 6-week training protocol progression.

Session Number	Session Length	Breathing Frequency	Session Number	Session Length	Breathing Frequency
1	3	20	12	13	22
2	4	20	13	14	24
3	5	20	14	15	24
4	5	20	15	16	24
5	6	22	16	17	24
6	7	22	17	18	44
7	8	22	18	18	26
8	9	22	19	19	26
9	10	22	20	20	26
10	11	22	21	20	26
11	12	22			

Length of the session in [minutes], breathing frequency in [breaths * min^−1^].

**Table 2:** Differences in the analyzed variables before and after VIH in normoxia across a 6-week respiratory muscle training intervention.

Variable	Value Before	Value After	Bayes Factor (BF_10_)	Bayesian Error %	Interpretation
Week 1					
pH	7.426 ± 0.01	7.423 ± 0.00	0.336	0.004	Anecdotal evidence for H_0_
HCO_3_^−^ (mmol/L)	27.089 ± 1.2	26.956 ± 1.4	0.435	0.008	Anecdotal evidence for H_0_
pO_2_ (mmHg)	71.744 ± 6.2	81.667 ± 8.4	7.986	<0.001	Moderate evidence for H_1_
pCO_2_ (mmHg)	41.244 ± 1.5	41.478 ± 3.1	0.331	0.004	Anecdotal evidence for H_0_
Week 4					
pH	7.411 ± 0.00	7.409 ± 0.04	0.327	0.004	Moderate evidence for H_0_
HCO_3_^−^ (mmol/L)	26.386 ± 1.2	26.543 ± 1.5	0.397	0.004	Anecdotal evidence for H_0_
pO_2_ (mmHg)	67.029 ± 10.4	75.814 ± 6.6	1.596	<0.001	Anecdotal evidence for H_1_
pCO_2_ (mmHg)	43.178 ± 4.2	41.456 ± 4.3	0.490	0.010	Anecdotal evidence for H_0_
Week 6					
pH	7.417 ± 0.02	7.417 ± 0.03	0.322	0.004	Moderate evidence for H_0_
HCO_3_^−^ (mmol/L)	26.386 ± 1.2	26.543 ± 1.5	0.323	0.004	Moderate evidence for H_0_
pO_2_ (mmHg)	75.344 ± 5.5	85.578 ± 8.8	7.531	<0.001	Moderate evidence for H_1_
pCO_2_ (mmHg)	41.067 ± 2.8	41.189 ± 4.2	0.324	0.004	Moderate evidence for H_0_

**Values are mean ± standard deviation.** H stands for the statistical hypothesis (conventionally H_0_—no significant differences, conventionally H_1_—significant differences).

**Table 3:** Differences in the analyzed variables before and after VIH in severe hypoxia (4200 m asl).

Variable	Value Before	Value After	Bayes Factor (BF_10_)	Bayesian Error %	Interpretation
pH	7.414 ± 0.02	7.443 ± 0.03	6.304	<0.001	Moderate evidence for H_1_
HCO_3_^−^ (mmol/L)	24.156 ± 1.4	24.733 ± 1.3	0.836	0.023	Anecdotal evidence for H_0_
pO_2_ (mmHg)	46.906 ± 3.7	51.761 ± 7.6	2.984	<0.001	Anecdotal evidence for H_1_
pCO_2_ (mmHg)	38.294 ± 2.7	35.783 ± 4.5	1.349	0.024	Anecdotal evidence for H_1_

**Values are mean ± standard deviation**. H stands for the statistical hypothesis (conventionally H_0_—no significant differences, conventionally H_1_—significant differences).

## Data Availability

The data supporting the findings of this study are available from the author upon request.
